# Calculating the design effect for a cluster stepped-wedge trial with varying cluster size; a case study from a trial in type 2 diabetes

**DOI:** 10.1186/1745-6215-16-S2-P124

**Published:** 2015-11-16

**Authors:** Nuzhat Ashra, Melanie Davies, Kamlesh Khunti, Laura Gray

**Affiliations:** 1 Diabetes Research Centre, University of Leicester, Leicester, UK; 2 Leicester Clinical Trials Unit, University of Leicester, Leicester, UK; 3 Department of Health Sciences, University of Leicester, Leicester, UK

## Background

Type 2 diabetes (T2DM) is a serious chronic disease which can be improved with education. Only 6.0% of people with T2DM are offered education and only 1.6% attend.

## Methods

We have designed a cluster trial with a stepped-wedge design (SWD) to test an intervention to increase the uptake to education. This study will include general practices of varying size. There is no published guidance on powering a cluster SWD with varying cluster size. We present the method used to estimate the design effect (DE) which combines the method for calculating the sample size required for a cluster SWD by Woertman et al. with a method for taking into account varying cluster size in a standard cluster trial by Eldridge et al.

## Results

We assume a median practice size of 347, IQR 201-678, which gives a Coefficient of Variation (CV) of 119.25/348=0.34. Using the method of Woertman gives a DE of 1.41; this doesn't take into account variation in cluster size. Using the method in Eldridge with a CV of zero (no variation in cluster size) gave DE of 18.35; replacing CV with 0.34 gave DE of 20.36. Hence variation in cluster size inflates DE by 2.01 (20.36-18.35). The total DE therefore when taking into account the SWD and variation in cluster size was 3.42 (1.41+2.01).

## Conclusion

We present a pragmatic way of calculating the DE for cluster SWD with variation in cluster size. Future work should focus on the impact of cluster size variation.

